# Profiling Prostate Cancer Therapeutic Resistance

**DOI:** 10.3390/ijms19030904

**Published:** 2018-03-19

**Authors:** Cameron A. Wade, Natasha Kyprianou

**Affiliations:** 1 Departments of Urology, University of Kentucky College of Medicine, Lexington, Kentucky, KY 40536, USA; cameron.wade@uky.edu; 2 Department of Molecular and Cellular Biochemistry, University of Kentucky College of Medicine, Lexington, Kentucky, KY 40536, USA; 3 Department of Toxicology & Cancer Biology, University of Kentucky College of Medicine, Lexington, Kentucky, KY 40536, USA

**Keywords:** epithelial plasticity, androgen receptor, tumor landscape, metabolic changes

## Abstract

The major challenge in the treatment of patients with advanced lethal prostate cancer is therapeutic resistance to androgen-deprivation therapy (ADT) and chemotherapy. Overriding this resistance requires understanding of the driving mechanisms of the tumor microenvironment, not just the androgen receptor (AR)-signaling cascade, that facilitate therapeutic resistance in order to identify new drug targets. The tumor microenvironment enables key signaling pathways promoting cancer cell survival and invasion via resistance to anoikis. In particular, the process of epithelial-mesenchymal-transition (EMT), directed by transforming growth factor-β (TGF-β), confers stem cell properties and acquisition of a migratory and invasive phenotype via resistance to anoikis. Our lead agent DZ-50 may have a potentially high efficacy in advanced metastatic castration resistant prostate cancer (mCRPC) by eliciting an anoikis-driven therapeutic response. The plasticity of differentiated prostate tumor gland epithelium allows cells to de-differentiate into mesenchymal cells via EMT and re-differentiate via reversal to mesenchymal epithelial transition (MET) during tumor progression. A characteristic feature of EMT landscape is loss of E-cadherin, causing adherens junction breakdown, which circumvents anoikis, promoting metastasis and chemoresistance. The targetable interactions between androgens/AR and TGF-β signaling are being pursued towards optimized therapeutic regimens for the treatment of mCRPC. In this review, we discuss the recent evidence on targeting the EMT-MET dynamic interconversions to overcome therapeutic resistance in patients with recurrent therapeutically resistant prostate cancer. Exploitation of the phenotypic landscape and metabolic changes that characterize the prostate tumor microenvironment in advanced prostate cancer and consequential impact in conferring treatment resistance are also considered in the context of biomarker discovery.

## 1. Introduction

Prostate cancer is the most frequently diagnosed cancer and the third leading cause of cancer deaths in males with an estimated 29,430 deaths in the United States for 2018, behind only digestive system and respiratory system cancers. There is an estimated incidence of 164,690 cases of prostate cancers in the United States for 2018 [1]. These cases account for approximately 19.2% of all estimated new cases of cancer in males in the United States. The incidence trend of prostate cancer between 2004 and 2013 showed a significant decrease of −4.8%, and a −8.6% change between 2009 and 2013. These averages are greater in magnitude than the trends in all site cancers for males of −1.6% and −2.9%, respectively [1]. The five-year survival for patients with non-metastatic prostate cancer is 98.9% (measure between 2005 and 2011) but patients with metastatic prostate cancer on initial diagnosis (4% of prostate cancer patients on diagnosis) had only a 28.2% five-year survival rate [1].

Almost all cases of patients with prostate cancer will progress to castration resistance, indicated by increasing serum levels of prostate specific antigen (PSA) despite castrate levels of testosterone and progress to metastases [2]. 10% to 20% of prostate cancers progress to castration resistant prostate cancer (CRPC) within 5 years of diagnosis, and 84% of newly diagnosed CRPC have metastases [2,3]. The median survival of patients following diagnosis of castration resistance ranges between 15 and 36 months [4]. Epidemiologic profiling of CRPC has been challenging to determine due to the lack of standardized diagnostic models, reporting methods for CRPC, and inconsistent terminology (castration-resistant, hormone refractory, and androgen independent are all used to describe CRPC). ICD-10 codes indicating CRPC were published for “hormone sensitivity status” and “rising PSA following treatment for malignant neoplasm of the prostate” in October of 2016 and these data may contribute to longitudinal epidemiologic information on CRPC. The estimated incidence of mCRPC in 2009 is 36,100 and for 2020 projected to 42,970. All-cause prostate cancer mortality from the same model was estimated at 219,360 for 2020 with mCRPC accounting for 19.5% (approximately 42,680) of these deaths [2]. Improvements to prostate cancer standards of care and 5-year survival following prostate cancer diagnosis have likely increased incidence of CRPC.

## 2. Standards of Care for Diagnosis and Treatment

### 2.1. Diagnosis of Prostate Cancer

Prostate cancer screening includes a digital rectal exam or a serum PSA test. Conventionally, 4.0 ng/mL or lower serum PSA was considered “normal”, and higher values indicate an increased risk of prostate cancer. Randomized trials of the PSA screening method have demonstrated comparable rates of prostate cancer among patients with less than and greater than 4 ng/mL [5]. Additionally, serum PSA may also fluctuate with prostatitis, urinary tract infections, previous prostate biopsy or surgery, and some drugs, including finasteride and dutasteride which lower serum PSA [6]. As a result, the National Cancer Institute recommends PSA screening be used based on patient risk factors and age. Prognostic scoring of prostate tumor biopsy specimens is evaluated by the Gleason system. Gleason scores range from 2 to 10 with higher scores representing a worse prognosis [7]. Scoring requires a biopsy of prostate tissue and is based on 5 different distinct structural phenotypes graded 1–5. A grade is assigned to the most prominent cell morphology, then added to the next highest grade structural phenotype (1–5) seen on histology. In a 2005 Gleason grading consensus conference it was decided that a Gleason score of 2 should be referred to as pre-cancerous adenosis, and that low-grade cancerous scores range from 3 to 4 [8]. The recent efforts towards personalized markers of prostate cancer diagnosis and prognosis, have established that Gleason score is highly associated with individual markers of cancer progression that help with clinical decision towards a course of treatment [8,9].

Additionally, the prostate intraepithelial neoplasia (PIN) phenotypes is assigned to a pre-cancerous phenotype characterized by prominent epithelial nucleoli in an otherwise normal glandular duct. High-grade PIN (HGPIN) is characterized by four distinct morphological patterns and is an indicator of risk for progression to malignancy [10,11,12]. Both Gleason scores and HGPIN markers are associated with similar genetic markers and suggest that genotyping of prostate cells with precancerous phenotypes may aid in early intervention and prevention of an invasive phenotype [13]. HGPIN prostate epithelium has been characterized by increased nearby microvasculature density when compared to normal glandular epithelium, suggesting the significance of angiogenesis in the development of premalignant HGPIN phenotype [12].

### 2.2. Treatment Strategies of Metastatic Prostate Cancer

Exploitation of the androgen sensitivity of prostate cancers for the clinical benefit of patients with prostate cancer was first defined by Charles Huggins et al. in 1941, in a classical study that showed a relationship between castration-induced androgen depletion and regression of clinical symptoms [14]. Mechanistically, the androgen receptor (AR) will dimerize and increase transcriptional activity and promote prostate epithelial proliferation when bound by testosterone or dihydrotestosterone (DHT) in the prostate. Testosterone is enzymatically converted to DHT intracellularly by 5-α reductase enzyme activity, resulting in higher circulating serum testosterone and DHT accumulation in tissues [15]. DHT binding of AR results in a 10-fold increase in transcriptional activity when compared to testosterone binding of AR [16].

Castration-induced androgen deprivation therapy (ADT) inhibits the action of testosterone and DHT on the AR signaling axis by keeping total serum testosterone below 50 ng/dL. ADT is the first-line suggested therapy for hormone naïve prostate cancer with or without metastases [17]. Estrogen therapy resulted in cardiovascular toxicity and is not indicated for use at high doses [18]. Gonadotropin-releasing hormone (GnRH) agonist therapies provided a less-toxic, reversible alternative to orchiectomy and estrogen therapy. GnRH agonist and AR antagonist ADT remain the first-line treatment for prostate cancers, with median response duration of only 18 months [3]. The relatively short response to ADT of approximately 1.5 years provides a clinical challenge to expand the duration of efficacy for first line prostate cancer therapies. The indicated first line therapies fail to increase 5-year year disease-free survival and at present, provide only a temporary patch that ultimately results in progression the resistance.

Among patients who initially present with androgen-sensitive prostate tumors that have already progressed to metastases, the first line therapy is ADT. Clinically, for patients with non-metastatic CRPC, a rapidly increasing PSA despite ADT is a known risk factor for metastases [16,19]. Hormone-naïve prostate cancer with markers of early metastasis, but lacking symptomatic and radiologic evidence of metastasis, the indicated course of clinical action therapy remains ADT with active surveillance. Second line treatments are indicated for ADT resistant mCRPC and include chemotherapy, second-line hormones and immunotherapy.

Docetaxel with prednisone is the indicated chemotherapeutic treatment for chemotherapy-naïve mCRPC patients. Docetaxel was approved by the FDA in 2004 as a combination drug with prednisone demonstrating a 2.5-month median survival benefit (total 19.2 months post-castration resistance) in CRPC patients being treated with mitoxantrone and prednisone (MP), the previous standard of care [20,21]. Docetaxel is a taxane that functions by inhibiting microtubule formation and blocking cell division, as well as disruption of AR signaling by inhibiting androgen-dependent AR nuclear translocation. Cabazitaxel, the second–line taxane chemotherapy has a similar mechanism of action and is indicated for docetaxel-resistant CRPC and demonstrates a 2.4-month median survival benefit (15.1 months total) when compared to other docetaxel resistant patients treated with MP [22,23]. Cabazitaxel is characteristically more toxic than MP and there is significantly larger risk of drug-related deaths, neutropenia, diarrhea, and febrile neutropenia when compared to MP [2,24].

The second-generation antiandrogens may prolong survival in patients with chemotherapy naïve mCRPC and docetaxel pretreated populations [3]. Abiraterone acetate is an androgen synthesis inhibitor that functions by irreversibly inhibiting CYP17A and is administered with prednisone. A double-blind placebo-controlled phase 3 study administered abiraterone acetate plus prednisone to patients with mCRPC that showed tumor progression after docetaxel treatment. Median survival for the abiraterone group was 4.6 months longer than the placebo in patients with mCRPC [2,3]. Enzalutamide is another androgen signaling inhibitor indicated for patients with mCRPC that prolonged survival in patients with mCRPC by a median of 4.8 months when compared to a placebo [25]. Enzalutamide competitively binds the testosterone/DHT receptor on AR, blocking translocation of AR to the nucleus, as well as DNA-binding of nuclear AR [26].

A newly FDA approved therapy, apalutamide, is indicated for patients who have CRPC, without radiographic or clinical signs of metastases. Apalutamide is a non-steroidal competitive inhibitor of AR used concurrently with ADT for patients with high-metastatic risk CRPC (PSA of ≥8 ng/mL or PSA doubling time of ≤10 months). Apalutamide recently demonstrated a 2.5-fold increase in median metastases-free survival when compared to placebo in a phase-3 trial (40.5 months for the apalutamide group versus 16.2 months for the placebo group) [27]. Like enzalutamide, apalutamide competitively binds the testosterone/DHT receptor of AR, therefore preventing translocation and DNA-binding of AR [28]. These results indicate that metastases may be postponed and may potentially eliminate the need to passively survey tumors that are “silently” progressing to aggressive metastases and provides clinicians an option for treatment prior to metastases.

Cellular immunotherapy is indicated for asymptomatic mCRPC and utilizes host immune system to stimulate a T-cell response against the prostate cancer. The process involves isolating patient peripheral blood antigen-presenting mononuclear cells and exposing the cells to recombinant prostatic acid phosphatase (PAP) antigen in vitro in the presence of granulocyte colony-macrophage stimulating factor (GM-CSF). PAP is commonly expressed in prostate cancers of all Gleason scores (GS), but decreases as score increases, one study showed 100% immunoreactivity among GS 6 and 7 tumors but decreased to 61% and 78.2% among GS 9 and 10 tumors, respectively. Therefore, PAP was chosen as the antigenic immune stimulator for sipuleucel-T, the first cellular immunotherapy approved by the FDA in 2010 [28]. The sipuleucel-T autologous PAP antigen-specific monocytes are infused three times in 2-week intervals and have shown a 33% reduction in risk of death with a 4.3-month median increase in survival time when compared to mCRPC patients receiving placebo [11].

## 3. Contributors to Therapeutic Resistance in Advanced Prostate Cancer

### 3.1. The Androgen Receptor (AR)

Patients with CRPC commonly have AR gain-of-function mutations that permit tumor cell growth in the absence or near-absence of androgens by increased ligand responsiveness, AR overexpression, and constitutive activation of unbound AR, resulting in an androgen signaling axis that is not mitigated by therapeutic repression of androgens [3,29]. In vitro studies of castration resistant prostate cancer cell lines displayed mutations of the AR ligand binding domain (HΔY874, TΔA877) that may increase tumor responsiveness to hydrophobic biomolecules including the original AR ligands testosterone and DHT, as well as novel ligands such as β-estradiol that entirely circumvent the tumor dependence on androgens. In vitro CRPC cells have also demonstrated increased AR expression, a characteristic found in 30% of clinical CRPC cases, or expression of constitutively active AR [16,30,31]. AR transcriptional coregulatory proteins (co-activators/co-repressors) also play a role in the progression to therapeutic resistance. In vitro studies have shown that prostate cancer cells grown in the presence of high doses of DHT displayed at least two-fold increases in the transcriptional activity of the genes *AIB1*, *CBP*, *MAK*, *BRCA1* [31,32,33]. AIB1 (Amplified in Breast Cancer 1) acts as a steroid receptor coactivator and is commonly overexpressed in primary breast carcinomas [33]. MAK (Male Germ Cell-Associated Kinase) acts as a coactivator of AR and an androgen-independent intracellular increase of *MAK* in prostate cancers may mediate androgen independence [34]. CBP (CREB binding protein) is another transcription factor that may increase the AR expression to promote castration resistance. Breast Cancer Protein 1 (BRCA1) is a well-known hormonally induced AR coactivator. Additionally, β-catenin, a protein associated with cell-cell adhesion as a part of E-cadherin, displayed decreased expression in cell lines with induced AR overexpression [35]. Loss of β-catenin expression is a potential contributor to metastases in CRPC by reducing cell-cell adhesions of de-differentiated epithelial cells and promotion of EMT [33].

### 3.2. Tumor Suppressor Action

A wealth of evidence has fueled the significance of loss of tumor suppressor genes such as *p53* and *pTEN* in prostate cancer initiation, progression and therapeutic resistance in advanced disease [36]. Commonly associated defects in tumor suppression and CRPC are *p53*, *pTEN*, ETS-related gene (*ERG*), and *BCL-2*. p53 protein has a well-defined role in cell-cycle maintenance in response to stressors such as DNA damage and oncogene expression, and a role in AR expression. p53 responds to intracellular stressors such as DNA breaks, UV radiation induced lesions, and hyper-proliferation by acting as a transcription factor for p21, which inhibits cyclin-dependent kinase (CDK) and arresting the cell in G1 [37]. Other functions of p53 include pro-apoptotic signaling, senescence, DNA repair, and differentiation, but the majority of p53 cell responses end in apoptosis [38].

Loss of pTEN progresses prostate cancers to CRPC by activation of the phosphatidylinositol 3-kinase (PI3K)/protein kinase-B (AKT) pathway. In normal cells PI3K activates phosphatidylinositol 3,4,5-triphosphate (PIP_3_) via phosphorylation, and PIP_3_ is degraded by pTEN to maintain homeostasis. In cases of partial or complete pTEN loss, undegraded PIP_3_ will downstream activate AKT, resulting in Bcl-2-associated death promotor (BAD) protein and caspase 9 inhibition, ultimately decreasing apoptosis, inhibition of forkhead box protein O4 (AFX) transcription factor, reducing expression of cell cycle regulator p27, and activate FKBP-12-rapamycin associated protein (FRAP)/mammalian target of rapamycin (mTOR) which induce translation of cyclin D1 to promote cell cycle progression [38]. Increased AKT expression is common in prostate cancers and is associated with poor prognosis, therapeutic resistance, and serves as an independent biochemical indicator of recurrence in prostate cancers [39]. Homozygous and heterozygous loss of the *pTEN* gene has been reported in up to 13–15% of local prostate tumors, and 30–39% of metastatic cases [10]. In one study, almost half of CRPC patients presented with partial or complete pTEN loss [40,41,42]. In a study of *ERG* and multi-phase prostate cancers (ADT treated cancers, CRPC, and mCRPC), expression was associated with loss of pTEN in prostatectomy and local CRPC patients. Loss of pTEN in the cohort was only associated with shorter progression-free survival only in *ERG* expressing patients, indicating the association between the two transcription factors and tumor progression. Most patients also displayed loss of p53 [43]. A recent study by Yang et al. demonstrated that loss of pTEN in human prostate cancer cells promotes activation of an AKT-runt related transcription factor 2 (Runx2) signaling axis that induces expression of steroidogenesis genes *CYP11A1*, *CYP17A1*, and intratumoral androgen synthesis [44]. Furthermore, it was established that *pTEN* null mice were prone to augmented intratumoral steroidogenesis, as well as microenvironment remodeling. The effects of pTEN loss in mice were diminished in the mice with heterozygous *Runx2* deletion, or treatment with the CYP17A1 inhibitor abiraterone acetate [45].

### 3.3. Growth Factor Signaling

**Figure 1:**
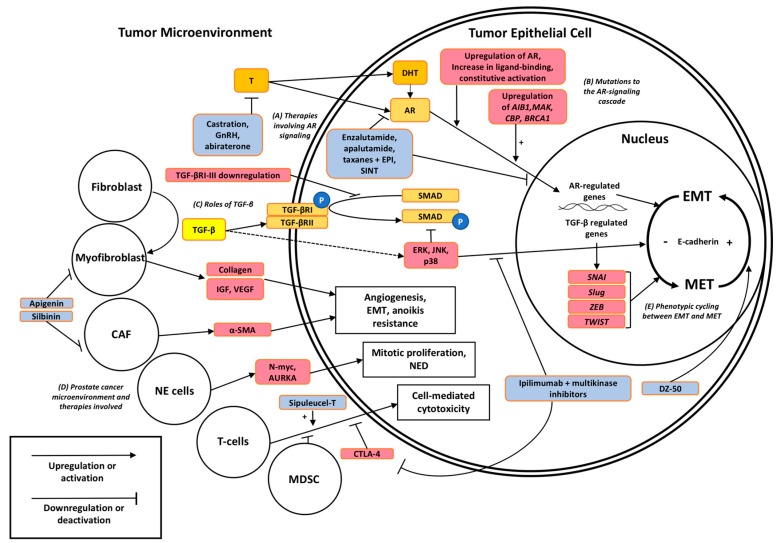
Signaling pathways contributing to therapeutic resistance in prostate cancer and their targetable interactions via EMT to MET interconversions. (A) First and second line antiandrogens (abiraterone and enzalutamide) target the AR signaling cascade by reducing testosterone production or inhibiting the binding site of AR and subsequent translocation to the nucleus, respectively. (B) Mutations in AR signaling promote transcriptional activation despite ADT. (C) TGF-β bi-functionally affects cell growth and differentiation through intracellular SMAD and non-SMAD signaling including MAP-kinases, loss of E-cadherin, and consequential changes in cell polarity. (D) Distinct cell types such as myofibroblasts, CAFs, neuroendocrine (NE) cells, and MDSCs (Myeloid-derived suppressor cells) within the microenvironment may navigate therapeutic resistance to antiandrogens and taxane chemotherapy by engaging ECM components, growth factors such as TGF-β, VEGF, IGF, mitotic promoters, and immune suppression. (E) Loss and gain of E-cadherin serves as a causative factor of cell polarity and biomarker of EMT, respectively, under the transcriptional repression of *SNAI*, *ZEB1*, and Twist-related protein (*TWIST*) (nuclear transcription factors). Color code: Orange/yellow: normal cellular signaling, red: promotors of therapeutic resistance, blue: existing or experimental therapies for prostate cancer.

Transforming Growth Factor-β (TGF-β): TGF-β is an intriguing cytokine with bifunctional roles in the regulation of the normal prostate growth, balancing the signaling interactions within the microenvironment, acting as tumor suppressor via the apoptosis induction in the early stages of tumorigenesis and switching to a metastasis promoter via effects on epithelial-mesenchymal transition (EMT) during tumor progression to metastasis. In normal prostate epithelium TGF-β largely signals through gene expression regulation and controls cell cycle and microenvironment through both Smad protein family (SMAD) and non-SMAD signaling pathways [45]. The first step in SMAD signaling is TGF-β binding to type II TGF-β receptor (TGF-βRII), a receptor with constitutively active serine/threonine kinase activity, and the ligand-receptor complexes transphosphorylate type I TGF-β receptors (TGF-βRI), ultimately forming a heterotetramer. Activated TGF-βRI molecules activate SMAD mediators via phosphorylation. The phosphorylated SMAD molecules complex with co-factors and travel to the nucleus to induce transcriptional changes. SMAD signaling can regulate transcriptional output of active genes and activate chromatin-repressed gene, this pathway is illustrated on Figure 1 [46,47]. TGF-βRIII is a third receptor type that acts as a co-receptor for TGF-β by binding G alpha interacting protein (GAIP) at the cell membrane and increasing binding affinity of TGF-β2 for the TGF-βR [48,49]. In healthy prostate or early prostate cancer, the TGF-β-SMAD signaling activates downstream apoptosis and inhibits cellular proliferation. Non-SMAD signaling by TGF-β leads to down regulation of c-Myc oncogene, resulting in upregulation of CDK inhibitors. c-Myc is a transcription factor that promotes cell growth and proliferation [50,51]. c-Myc downregulation by TGF-β is a key step in the inactivation of G_1_ CDK proteins driving cell proliferation. In particular, TGF-β induces expression of p15ink4b (p15), a cyclin-dependent kinase 4 inhibitor tumor suppressor protein, that functions to induce dissociation of cyclin-D1 from CDK and prevention of cyclin D1-CDK complex formation, arresting cells in G_1_.

Insulin-Growth Factor (IGF): IGF also emerges as regulator of EMT upon binding of IGF ligands, to IGF receptors IGF-IR and IGF-IIR, and consequential activation of pro-EMT cascade via AKT signaling which also upregulates the ZEB protein [52,53]. In a cross-talk mechanism, insulin growth factor binding protein-3 (IGFBP3) is involved in IGF signaling regulated by TGF-β. IGFBP3 is overexpressed in some cancers, and silenced in others [54,55]. In healthy cells, IGFBP3 regulates IGF-I and/or IGF–II by binding the molecules and preventing the proliferation cascades initiated by IGF-R activation. Overexpression of IGFBP3 results in excessive induction of *SNAI1*, *ZEB1*, and *ZEB2* gene transcription [56]. The upregulation of the ZEB protein promotes TGF-β-mediated EMT and enables a potential target for overcoming therapeutic resistance.

Vascular-Endothelial Growth Factor (VEGF): VEGF includes a family of factors, VEGF-A, VEGF-B, VEGF-C, VEGF-D, and placenta growth factor, that play a distinct role in promoting angiogenesis in human malignancies including prostate cancer. VEGF expression is induced by both androgens (by non-canonical androgen signaling) and hypoxic environments [54]. In prostate cancer VEGF promotes angiogenesis by binding VEGF-R2 (Flk-1) on the vascular endothelial lining to promote proliferation and vascular permeability, then organization of nascent capillary tubes into the tumor microenvironment via VEGF-R1 (Flt-1) [57,58,59]. Preclinical studies in androgen-sensitive prostate cancer xenografts demonstrated that ADT results in a significant of VEGF levels and subsequent androgen replacement led to upregulation of VEGF expression [60]. VEGF has been the target of diverse pre-clinical and clinical prostate cancer trials, but the therapeutic response and survival outcomes remain “a road less taken” to impair advanced disease and impact patients in clinical practice [59].

### 3.4. Metabolomic Changes

Genetic changes that progress prostate cancer are known to create unique metabolomic profiles that may be used as both a diagnostic and prognostic tool, as well as an investigative platform to identify new metabolic targets for novel therapeutics [61]. Thus, the increase in glycolysis that leads to increased lactate in most cancer types, known as the “glycolytic switch” or “Warburg effect” described by Otto Warburg in 1956, is detected in PI3K-driven prostate tumors [62]. Prostate cancer is metabolomically characterized by increases fatty acid metabolism by fatty acid synthase as well as an increase fatty acid uptake when compared to healthy prostate cells [63,64]. Further, higher concentrations of fatty acid synthase mRNA and protein are associated with higher Gleason score and as an independent predictor of bone metastasis [65,66,67]. Among *TMPRSS2-ERG* translocation positive samples, a characteristic mutation in prostate cancers, fatty acid oxidation related metabolites were significantly increased, namely cerebronic acid, 2-hydroxybehenic acid, and tricosanoic acid [67,68].

A metabolomic change unique to prostate cancer is the decrease in citrate concentration and increase in citrate metabolite secretion, mediated by the activation of the enzyme m-aconitase [67]. Zinc acts as an inhibitor of m-aconitase in healthy prostate tissue but decreases in concentration as the prostate undergoes neoplastic progression [69,70]. Decreases in the cellular concentration of glucose, mannose, maltose, and maltotriose is concurrent with the decrease in intracellular citrate and increase in fatty acid metabolites in TMPRSS2-ERG positive tumors, support contribution of this mutation as a key initiation event in the progression from early pre-malignant phenotypes such as PIN to malignant prostate cancer [70]. The increased use of fatty acid oxidation and citrate metabolism increases available ATP in prostate cancer [67] For prostate cancer cells treated with ADT, the concentrations of lactate and t-choline are expected to decrease, and can be monitored by magnetic resonance imaging (MRI) to observe any increases despite treatment [71]. In addition to tumor grade and aggressiveness, metabolomic markers may indicate castration resistance via steroid autogenesis and/or changes to the androgen signaling axis [72]. Similarly, genomic microRNA (miRNA) profiles of prostate cancer samples may be a way to take a profile “snapshot” of the metabolic tumor stage and provide both diagnostic and prognostic information [73]. In particular, the miRNAs miR-96 and miR-21 found in tumor tissues have been identified as indicators of castration resistance that are positively correlated with tumor grade [74,75]. miRNA changes mediate therapeutic resistance by a wide set of oncogenic interactions including resistance to apoptosis, inhibition of metabolic regulatory genes (namely *FOXO1*), and notably the upregulation of AR in prostate cancer, providing a direct link to castration resistance [73,76]. Choline phosphate and cysteine both serve as two strong metabolomic predictors of disease recurrence [72,77,78]. Thus, advanced knowledge of metabolomics and interpretation of data on metabolic alterations, will lead to the identification of critical metabolites, as novel signature biomarkers of prostate cancer progression and emergence of therapeutic resistance.

## 4. The Impact of Microenvironment on Prostate Tumor Progression

### 4.1. The Prostate Defines Its Niche in the Microenvironment

The human prostate gland is organized as a lumen surrounded by secretory luminal epithelium, basal, and neuroendocrine cells. Deep to the basal lamina is a fibromuscular stroma comprised of fibroblasts, myocytes, endothelial cells, autonomic nerve fibers, immune cells, and a collagen-rich extracellular matrix (ECM) [79]. In normal prostate tissues, detachment of epithelial cells from the ECM induces apoptotic cell death through a process called anoikis. The missing epithelial cells are subsequently replaced by proliferation of endogenous progenitor epithelial cells [80,81]. Anoikis and stromal factors normally maintain prostatic homeostasis, but in both tumorigenesis and progression to mCRPC the stroma may promote tumorigenesis through vascularization, anoikis resistant survival mechanisms, and EMT [82].

As summarized on Figure 1, the functional interactions between cancer-associated fibroblasts (CAFs), endothelial cells, lymphocytes, and cancer epithelial cells play an important role in progression to metastases by promoting angiogenesis, repair, and survival as a result of reactivity to TGF-β [83,84]. Stroma reactivity to TGF-β may progress cancer independence from existing prostate vasculature and establish an independent nutrient and waste exchange via the vascularization of the tumor [46,85]. TGF-β signaling has been shown to stimulate myofibroblast formation from existing prostate fibroblasts [81]. Myofibroblasts promote tumor progression by repairing and regenerating damaged tissue, secretion of ECM components such as collagen, and secretion of angiogenesis promoting growth factors [50].

Neuroendocrine (NED) prostate cancer is a rare clonally proliferated subtype of prostate adenocarcinoma that is pathologically evident as a small and round undifferentiated endocrine-paracrine modulating cell type on histologic staining. Clonal proliferation of NED from an existing prostate cancer is indicated by the prostate cancer specific gene rearrangement *TMPRSS2-ERG* in NED prostate cancer cases, that most commonly appears following hormonal therapy of hormone sensitive prostate cancer [86]. Metastatic NED prostate cancers are characterized by lytic bone lesions, castration resistance, more rapid progression to metastases, visceral metastases, prostatic enlargement, and low PSA in metastatic disease [87,88]. Neuroendocrine cells do not express AR or PSA, but have markers such as chromogranin A, synaptophysin (SYP), and neuron-specific enolase (NSE) that give NED prostate cancer a distinct biomarker profile and should cause suspicion in cases of metastatic CRPC with low serum PSA [89]. Genotyping of NED prostate cancer revealed gene amplifications found significantly higher than non-NED prostate cancer, *AURKA* and *MYCN*. *AURKA* encodes Aurora kinase A, a serine/threonine kinase involved in mitosis that acts as an oncogene when amplified or overexpressed. *MYCN* encodes N-Myc, a transcription factor common to nerve tissue not expressed in the prostate known to induce expression of Aurora kinase A [90]. Transfection of both *AURKA* and *MYCN* to non-NED prostate cell lines induced expression of the NED-specific markers SYP and NSE [91]. These results suggest that these genes may be responsible for the progression to the NED phenotype and provide a potential target for protein or RNA inhibitors to prevent metastases facilitating tumor microenvironments.

The tumor microenvironment facilitates therapeutic resistance by modification of stromal components to promote invasion, angiogenesis, and metastases. A characteristic change in the tumor microenvironment change in metastatic disease is the progression from fibroblasts, an abundant mesenchymal cell type in the extracellular matrix, to carcinoma-associated stromal cells (CAFs). Activated CAFs normally secrete alpha-smooth muscle actin (α-SMA) which acts as a chemical indicator of CAF expression and may play a role in facilitation of EMT of the prostate cancer when CAFs are constitutively activated in tumor states [88]. Both CAFs and myofibroblasts have repair-centric activities that promote tumor growth, empowering investigators with new therapeutic platforms exploiting the inhibition of stromal expression patterns that favor cell differentiation.

The novel drug DZ-50, a quinazoline derivative generated and characterized in our lab, has been shown to target the EMT dynamic by promoting the reverse process, MET. Molecular analysis revealed that DZ-50 causes reversion of EMT to MET in prostate cancer cells by engaging the IGF signaling [92]. DZ-50 inhibits the IGF signaling pathway by down-regulating gene expression of specific IGF-binding protein (IGFBP), IGFBP3, and thus antagonizing TGF-β1 mediated stabilizing of the EMT phenotype [93]. Drug-discovery and repurposing efforts exploit TGF-β signaling effectors reprogramming phenotypic changes that facilitate tumor progression and treatment resistance. Targeting the pathways that confer tumor progression by fostering the EMT landscape, will potentially lead to marginal increases in survival with current standards of care by deconstructing a microenvironment that sustains therapeutic resistance in individual tumors.

### 4.2. EMT Landscaping CRPC

The process of epithelial-mesenchymal transition (EMT), was first characterized as the dramatic extracellular changes that impact epithelial cell polarity and was classified as a distinct phenotypic pattern of tissue landscape in 1995 [93]. EMT in prostate cancer confers therapeutic resistance, invasive properties of the tumors and negatively impacts survival in prostate cancer patients [94]. Conversion of epithelial cells to mesenchymal cells involves profound structural changes, including loss of cell-cell adhesion, degradation of basement membrane, loss of cell polarity and the acquisition of migratory and invasive properties [95]. EMT is thus a critical and functionally convenient venue for epithelial-derived tumors to become invasive and metastasize [94,96]. EMT endows cells with migratory and invasive properties, induces stem cell properties, prevents apoptosis, and orchestrates metastasis [94].

E-cadherin is essential in the maintenance of epithelial cell-cell adhesions. Loss of E-cadherin in place of N-cadherin is a key phenomenon common to EMT [83,94,97]. E-cadherin is encoded by the *CDH1* gene on chromosome 16q and maintains cell-cell adhesion by Ca^2+^ dependent junctions involving the actin microfilaments α-and β-catenin [98]. Androgen deprivation can potentially result in E-cadherin transcriptional repression, which is sufficient to confer the EMT phenotype, and inducing the expression of N-cadherin in androgen-dependent prostate tumors [99,100]. High N-cadherin/E-cadherin ratio phenotypes are associated with highly invasive cancers. Further, N-cadherin expression and mRNA levels were increased in androgen-independent tumors in a castration environment [99,101]. Loss of epithelial-cell markers E-cadherin and β-catenin and gain of mesenchymal-cell markers N-cadherin and vimentin at the leading edge or invasive front of solid tumors are linked to metastatic progression [101,102,103]. These studies indicate N-cadherin as a potential target for therapeutics to prevent or mitigate metastatic phenotypes. Functionally it is the signaling activities of mesenchymal cells that facilitate migration and survival in an anchorage-independent, anoikis-defying mode [104,105]. In prostate cancer cells EMT is induced by TGF-β and/or androgens, with a threshold AR level determining the phenotypic outcome and invasive properties [97]. Transcriptional repression of E-cadherin by factors such as SNAI1/SNAI2, Slug, and TWIST1 block the expression of E-cadherin, disrupting the cell-cell junctions, and allows β-catenin to enter the nucleus and act as a regulator of EMT [84]. The reverse process of EMT, MET, provides metastatic CRPC the ability to differentiate into a phenotype that is better suited to colonize distant sites from the original tumor [96,106,107]. The processes of EMT and MET may occur cyclically in mCRPC cell lines and the variety of mCRPC phenotypes conferred by MET represents an effective therapeutic endpoint due to the loss of specific cellular targets [105,108].

### 4.3. TGF-β: Master Navigator of EMT

In advanced stage high grade prostate tumors, TGF-β serves as an inducer of EMT, by signaling de-differentiation, cell proliferation, and inhibition of apoptosis by non-SMAD (non-canonical) signaling pathways [105]. Loss of TGF-βR expression among tumor-stroma in various human malignancies has been associated with poor clinical outcomes [46,47]. In a retrospective study of TGF-βRII in colon carcinoma demonstrated that downregulation of the receptor is a strong independent prognostic indicator for survival, similar to lymph node metastases and vessel infiltration [109]. In prostate cell lines with induced TGF-βRIII knockdown, there was an associated increase in prostate CD133 expression [109]. In epithelial cells, the non-canonical TGF-β pathway engages mitogen activated protein kinase (MAPK), Rho-like GTPase, and PI3K/AKT as signaling effectors [110]. The MAPK signaling mechanism, upon TGF-β recruitment, leads to activation of rapidly accelerated fibrosarcoma (RAF) kinase/extracellular signal-related kinase (ERK), JNK/p38, and P13K/AKT, a set of MAPK proteins that bi-functionally induces EMT and blocks SMAD signaling [111]. TGF-β interacts with the AR signaling axis directly and indirectly to complete its opposing roles in the life cycle of the cell. In the presence of AR coactivators such as AR-associated protein 55 (ARA-55), AR can inhibit the up-regulatory effect of TGF-β on SMAD transcriptional activity. This evidence provides proof of principle that activated AR signaling in prostate tumor cells can counteract the tumor-suppressor function of TGF-β towards emergence of CRPC.

The reign of TGF-β as a bifunctional controller within the tumor microenvironment is perhaps most prominent in the late stages of progression to metastases. TGF-β2 is known to mediate the conversion of naïve prostate fibroblasts to CAFs expressing α-SMA [112]. As mentioned before, the prostate microenvironment is particularly reactive to TGF-β. Stroma CAFs, endothelial cells, lymphocytes, and cancer epithelial cells all appear to facilitate angiogenesis in response to TGF-β [85,113]. siRNA induced AR-knockdown CAFs demonstrated decreased TGF-β expression, indicating a relationship between the androgen signaling axis and TGF-β [81]. In mouse models of prostate tumorigenesis driven by aberrant TGF-β1 signaling there is increased collagenous micronodules, a lesion associated only with cancer and not benign glands [114]. The microRNA family miR-200 and zinc finger E-box-binding homeobox (ZEB) have been implicated in transcriptionally regulating the EMT to MET interconversions [115]. ZEB proteins transcriptionally repress E-cadherin and EMT is a consequence of ZEB overexpression [55]. In cells with hyper-methylated miR-200 loci demonstrate a stabilized ZEB/miR-200 molecular imbalance and maintaining the EMT phenotype [55]. The increased TGF-β1 secretion in the engineered *TWIST* overexpression cells correlated with increased SMAD activity and SMAD2 phosphorylation, an autocrine TGF-β-mediated maintenance mechanism for mesenchymal phenotypes [116].

## 5. Targeted Therapies to Overcome Therapeutic Resistance

Bioinformatics-driven studies focus on personalized marker discovery to predict treatment failure in individual prostate cancer patients. The emerging scenario defines the significance of cellular stroma microenvironment dynamics towards individualizing therapy with existing treatments, by acknowledging the stroma influence and co-evolution with cancer, particularly fibroblastic changes that facilitate the epithelium to de-differentiate [117]. As mentioned before, PSA doubling-time has been recognized as an indicator for risk of metastases [118]. Overall survival prognostic models for mCRPC patients that included lactate dehydrogenase (LDH), albumin, hemoglobin, PSA, and alkaline phosphatase as variables (among other performance markers) are highly predictive of patient survival [20,21]. Cytokines involved in tumor growth and metastases, interleukin 6 (IL-6) and tumor necrosis factor-α (TNF-α), are also important serum biomarkers with high prognostic value in prostate cancer patients [119,120]. Serum IL-6 is significantly increased in patients with CRPC when compared to values of healthy controls, patients with benign prostatic hyperplasia (BPH), and localized prostate cancer [121,122]. Patients showed decreased overall survival when serum values of both IL-6 and TNF-α are found above the 95th percentile values of healthy controls when compared to prostate cancer patients with values below the cutoff [123]. The use of transcriptomics and genomics to identify markers such as *AR* signaling genes, AR splice variants, *P13K/AKT* pathway genes, and other housekeeping genes, as well as somatic mutations in *FOXA1*, *SPOP*, *pTEN*, pathogenic mutations in *TP53*, and germline mutations in *BRCA2* or *ATM* has been most creative. Genetic testing on patients may suggest simultaneous regimens of ADT and docetaxel as first-line therapy when genotypic markers show high-risk profiles in localized tumors [122]. This provides a molecular basis to overcome ADT resistance in a subset of CRPC patients harboring AR mutations/variants, modulated intratumoral steroidogenesis, loss of DNA repair mechanisms, and changes in phenotypic landscape such as EMT and NED [124,125].

Compelling new insights into the combination strategies of novel AR-signaling inhibitors that bind the N-terminal domain (NTD) of AR, EPI-001/002, and taxane chemotherapy, have established in pre-clinical in vivo and in vitro models the synergistic action in the reversal of de-differentiated EMT phenotypic CRPC to a guided MET phenotype that is more conventionally susceptible to therapies [88]. As shown on Figure 1, the novel AR-NTD inhibitor sintokamide A (SINT) is effective in vivo at regressing CRPC xenografts and reducing PSA, with additive effects to EPI due to targetable interactions between AR-NTD and target STAT3 [126].

Myeloid-derived suppressor cells (MDSCs) play an important role in tumor evasion of the immune system, and increased MDSCs are correlated with serum PSA and metastases of prostate cancer [127]. Anti-cancer multikinase inhibitors such as cabozantinib and BEZ235 are targeted at metastatic-infiltrating CRPC and display minimal activity against tumors. In addition to circulating MDSCs, regulatory inhibition of cytotoxic T-cells by cytotoxic T-lymphocyte-associated protein 4 (CTLA4) downregulates the immune response by inactivating the cell-mediated immune response. Therapies such as ipilimumab target the CTLA-4 cascade as an inhibitor of the regulatory cascade. Trials of ipilimumab therapy in mCRPC have failed to demonstrate efficacy [128,129]; however, in trials with combination therapies (anti-MDSC and immunomodulatory), patients with either localized disease or mCRPC demonstrated robust responses [130,131]. These studies support a promising clinical benefit surrounding the combination regimens of immunomodulatory drugs and anti-MDSCs as effective in overcoming therapeutic resistance (Figure 1).

The chemo-preventive agent silibinin is used as a preventative therapy for patients with naïve prostate cancer to impair disease progression to mCRPC. Silibinin inhibits induction of mesenchymal phenotype (CAFs) and overexpression of α-SMA by blocking TGF-β2 expression [132]. Similarly, treatment with apigenin, a naturally occurring flavone decreases the TGF-β1 induced expression of VEGF in mice and reduces the fraction of α-SMA myofibroblasts in ex vivo lung cancer biopsies [85]. The target sites for both silibinin and apigenin are shown on Figure 1. Other therapies including monoclonal antibody against VEGF, bevacizumab, that reduced tumor volume by targeting tumor vascularity, failed to demonstrate significant improvement in overall survival for patients already on taxane chemotherapy with prednisone [113,133].

## 6. Conclusions

In the year 2018, molecular technology and large data processing provide great new platforms for understanding to complexity and heterogeneity of prostate tumors and developing strategies to prevent, postpone, or mitigate the migratory and invasive phenotypes of prostate cancer, beyond the AR signaling. Exploitation of EMT regulatory proteins as distinct phenotypic markers of tumor progression, as well as novel therapeutic targets including cellular processes mediated by the TGF-β non-SMAD signaling family that facilitate a tumor-promoting microenvironment, will lead to precision diagnosis and optimized combination strategies to impair metastatic and therapeutically resistant disease. Utilizing the distinct markers that arise from the microenvironment modifiers such as the neuroendocrine cells or CAFs, may provide a clinical edge to tumor progression provided available therapeutic targets for the identified stromal pathways. The tumor microenvironment consisting of myofibroblasts, CAFs, neuroendocrine cells, and MDSCs has been under a low-profile pursuit as potential therapeutic targets/platforms, despite compelling evidence as to their functional contribution to the phenotypes driving tumor progression to metastases and emergence of therapeutic resistance (Figure 1).

## Abbreviations

AR	androgen receptor
CRPC	castration-resistant prostate cancer
PSA	prostate specific antigen
EMT	epithelial-mesenchymal-transition
MET	mesenchymal epithelial-transition
TGF-β	transforming growth factor-β
DHT	dihydrotestosterone
ADT	androgen deprivation therapy
CDK	cyclin dependent kinases
ECM	extracellular matrix
MDSCs	Myeloid-derived suppressor cells
CAFs	Cancer-associated fibroblasts
VEGF	Vascular endothelial growth factor
IGF	insulin growth factor
IGFBP3	Insulin growth factor binding protein-3
